# 

**Published:** 1849-01

**Authors:** 


### CONTENTS OF NO. I.

### ORIGINAL COMMUNICATIONS.

### ESSAYS AND CASES.

ART. I.—On the Progress of Etherization. By LUNSFORD P. YANDELL, M.D., Professor of Chemistry and Pharmacy in the University of Louisville.......................... 1

ART. II.—A Case of Colic attended with obstinate Constipation; Stercoraceous Vomiting; Recovery. By JOHN P. DROMGOOLE, M. D., of Rutherford County, Tennessee.......... 37

### REVIEWS.

ART. III.—The Principles and Practice of Medicine. By JOHN W. HOOD, M. .................................... 40

ART. IV.—Introductory Lectures. An Introductory Lecture delivered in the Medical College of Georgia, at the opening of the annual session, Nov. 6th, 1848. By JOSEPH A. EVE, M. D., Professor of Obstetrics and Diseases of Women and Infants.

An Introductory Lecture on the Coinciding Tendencies of Medicines. By JARED P. KIRTLAND, M. D., Professor of Theory and Practice of Medicine, and Physical Diagnosis in the Medical Department of the Western Reserve College at Cleaveland, Ohio.

An Introductory Lecture delivered to the Class of Midwifery and Diseases of Women and Children, in Jefferson Medical College, October 18th, 1848. By CHARLES D. MEIGS, M. D. ........................................ 40

Art. V.—Principles of Zoology: Touching the Structure, Development, Distribution and Natural Arrangement of the Races of Animals, living and extinct; with illustration. For the use of Schools and Colleges. Part I. Comparative Physiology. By Louis Agassiz and Augustus A. Gould.......................................53
Art. VI.—A Manuel of Auscultatlon and Percussion. By MM. Barth and Roger—translated, with additions, by Francis G. Smith, M. D.......................................55

SELECTIONS FROM AMERICAN AND FOREIGN JOURNALS.

Typhus and Typhoid Fevers.......................................67
A New Febrifuge.......................................83
Accidental Poisoning with bad Calomel in France.......................................84
Medical Profession in Paris.......................................84

ORIGINAL INTELLIGENCE.

Medical Schools.......................................84
Extension of the Lecture-Term.......................................88
Sectional Medicine.......................................89
Chloroform in Traumatic Tetanus.......................................89
Fevers in Alabama.......................................90
Progress of Cholera.......................................90
Lawson on Cholera.......................................91
Convention of Western Medical Schools.......................................91
Webster's Dictionary.......................................91
Influenza.......................................92
A New Work on Parturition.......................................92

